# Spatial diversity of the skin bacteriome

**DOI:** 10.3389/fmicb.2023.1257276

**Published:** 2023-09-19

**Authors:** Marcos Pérez-Losada, Keith A. Crandall

**Affiliations:** Department of Biostatistics and Bioinformatics, Computational Biology Institute, Milken Institute School of Public Health, The George Washington University, Washington, DC, United States

**Keywords:** 16S rRNA, bacterial diversity, bacteriome, functional diversity, skin microbiome

## Abstract

The bacterial communities of the human skin impact its physiology and homeostasis, hence elucidating the composition and structure of the healthy skin bacteriome is paramount to understand how bacterial imbalance (i.e., dysbiosis) may lead to disease. To obtain an integrated view of the spatial diversity of the skin bacteriome, we surveyed from 2019 to 2023 five skin regions (belly button, behind ears, between toes, calves and forearms) with different physiological characteristics (dry, moist and sebaceous) in 129 healthy adults (579 samples – after data cleaning). Estimating bacterial diversity through 16S rRNA metataxonomics, we identified significant (*p* < 0.0001) differences in the bacterial relative abundance of the four most abundant phyla and 11 genera, alpha- and beta-diversity indices and predicted functional profiles (36 to 400 metabolic pathways) across skin regions and microenvironments. No significant differences, however, were observed across genders, ages, and ethnicities. As previously suggested, dry skin regions (forearms and calves) were more even, richer, and functionally distinct than sebaceous (behind ears) and moist (belly button and between toes) regions. Within skin regions, bacterial alpha- and beta-diversity also varied significantly for some of the years compared, suggesting that skin bacterial stability may be region and subject dependent. Our results, hence, confirm that the skin bacteriome varies systematically across skin regions and microenvironments and provides new insights into the internal and external factors driving bacterial diversity.

## 1. Introduction

The skin is the largest organ in the human body with an average surface area in adults of 30 m^2^ (Gallo, 2017). It has a protective role, acting both as a physical barrier against environmental factors and as an immunological barrier, reducing the effects of injuries and infections. The skin also has a thermoregulatory function; preventing water loss, enabling temperature regulation and supporting vitamin D synthesis (Grice et al., 2009; Grice and Segre, 2011; Byrd et al., 2018; Cundell, 2018).

To a large extent, skin’s physiology and homeostasis is impacted or maintained by the skin bacteriome – defined here as the collection of all bacteria living on our skin. The bacteriome protects us against invading pathogens by training and communicating with our immune system, and is involved in wound healing and breaking down natural products (Scharschmidt and Fischbach, 2013; Belkaid and Segre, 2014; Grice, 2015; Boxberger et al., 2021). The skin bacteriome harbors millions of bacteria, rivaling in composition and diversity the gut microbiome (Costello et al., 2009; Grice et al., 2009; Boxberger et al., 2021).

Typically, the bacterial community composition of the skin of healthy individuals is dominated by members of the phyla Actinobacteria, Firmicutes, Proteobacteria and Bacteroidetes. The composition and structure of the healthy skin microbiome vary between people depending on intrinsic and extrinsic factors (Grice and Segre, 2011; Cundell, 2018; Dimitriu et al., 2019; Boxberger et al., 2021; Skowron et al., 2021). Intrinsic factors include, for example, skin biogeography or physiology, ethnicity, gender and age; while extrinsic factors may include lifestyle, hygiene routine, cosmetics, antibiotics, geographical location, climate and seasonality (Dimitriu et al., 2019; Skowron et al., 2021). Among those factors, skin biogeography and associated physiology have been suggested as the main driver of skin microbial variation; microenvironments with comparable physiological characteristics tend to harbor similar bacterial communities, while those physiologically distinct, sebaceous (e.g., head locations), dry (e.g., forearms and legs) and moist (e.g., navel, toe web space), vary in bacterial membership and abundance (Grice et al., 2009; Oh et al., 2014; Byrd et al., 2018; Cundell, 2018; Skowron et al., 2021). *Cutibacterium* (formerly known as *Propionibacterium*) species seem to predominate in sebaceous sites, *Corynebacterium*, β-Proteobacteria and *Staphylococcus* species in moist sites, while a mixed population of those bacteria and Flavobacteriales occur in dry sites (Grice et al., 2009; Oh et al., 2014). Similarly, these two studies have also shown that sebaceous sites were less even and rich than moist and dry sites.

However, to what extent bacterial skin profiles are consistent across individuals remains to be seen. Similarly, few studies have explored the longitudinal stability of the skin microbiome (Costello et al., 2009; Grice et al., 2009; Oh et al., 2016), although they seem to indicate that some sites are largely stable over time, while others show appreciable variation. Even less understood is the functional diversity of the skin bacteriome and its variation across skin regions (Kong, 2011; Schommer and Gallo, 2013; Oh et al., 2014; Byrd et al., 2018; Wang et al., 2021). Recent research has reported that most metabolic pathways are not evenly distributed across body sites (Oh et al., 2014) as previously suggested (Human Microbiome Project, 2012), with functional capacity driven primarily by skin biogeography and individuals.

Therefore, further research is needed to describe the “normal” skin bacteriome of healthy individuals. A better understanding of the bacteria inhabiting distinct physiological sites may provide insights into the delicate balance between skin health and disease and the internal and external factors leading to dysbiosis. Toward this end, we used 16S amplicon high-throughput sequencing and metataxonomics to characterize the bacteriomes of 129 healthy individuals across five skin locations with different physiological properties. Subject individuals were part of an academic course in genomics (either undergraduate or graduate) and their bacteriome sampling and characterization was part of a project-based learning effort for the class (Pérez-Losada et al., 2020). As a consequence, the subjects were all healthy individuals with a relatively narrow and young age distribution. In this cross-sectional study, we investigated how bacterial taxonomic and functional profiles are partitioned across human skin regions, habitats, genders, ethnicities and years.

## 2. Materials and methods

### 2.1. Cohort

This is a cross-sectional study where participants were sampled once and the recruitment period lasted 5 years with sampling occurring in four of those 5 years. We recruited healthy undergraduate and graduate students from the Milken Institute School of Public Health at The George Washington University (Washington, DC, USA) every January in 2019, 2020, 2022, and 2023 – we skipped 2021 because of SARS COVID-19 restrictions. Students self-reported not having any skin diseases and not taking antibiotics or using topical steroids for at least the last month before skin sampling (exclusion criteria). This study was approved by the George Washington University Committee on Human Research, Institutional Review Board in 12/20/2018, IRB# 180703. All methods were performed in accordance with the relevant guidelines and regulations. Written consent was obtained from all adult participants using the informed consent documents approved by the George Washington University Committee on Human Research. This study was part of a project-based learning component of a course in Public Health Genomics at George Washington University, Washington, DC, USA (Pérez-Losada et al., 2020).

### 2.2. Sampling

A total of 129 students from the Washington DC area participated in the study (Supplementary Table S1). All students self-swabbed five regions or sites of their skin: belly button (BB), behind both ears (BE), between toes of both feet (BT), both calves (CA) and both forearms (FA). These five regions comprise three habitats or microenvironments with unique physiological properties: sebaceous or oily (BE), moist (BB and BT), and dry (CA and FA) (Grice et al., 2009; Grice and Segre, 2011; Byrd et al., 2018); they are also characteristically affected by dermatologic disorders where microbes have been implicated in disease pathogenesis (Grice et al., 2009). We followed the recommendations and sampling protocols used in the Human Microbiome Project (2012) and Kong (2016). Briefly, each region was sampled twice for 30 s using two sterile catch-all swabs moistened with SCF-1 solution (Tris-EDTA and 0.5% Tween-20). Both swabs were placed in the same Eppendorf tube containing ZymoBIOMICS™ Lysis Solution and processed together directly after sampling – see below.

### 2.3. 16S rRNA amplicon sequencing

Total DNA was extracted from swabs using the ZymoBIOMICS™ DNA Miniprep Kit D4300. All extractions yielded >2 ng/μL of total DNA, as indicated by NanoDrop 2000 UV–Vis Spectrophotometer measuring. DNA extractions were prepared for sequencing using the metataxonomic protocol (Marchesi and Ravel, 2015) described in Kozich et al. (2013). The V4 hypervariable region of the bacterial 16S rRNA gene was amplified using primers 515F – GTGCCAG CMGCCGCGGTAA and 806R – GGACTACHVGGGTWTC TAAT. Amplicons were then sequenced on the Illumina MiSeq platform at the George Washington University Genomics Core using 2×250 base pair, paired-end sequencing. While the V4 region has been shown to cover most of the described human bacterial diversity, it is also known that V4-specific primers are biased against some abundant skin bacteria, particularly *Cutibacterium* (Kong, 2016; Meisel et al., 2016). However, V4 primers can detect taxa that are underrepresented in skin microbiome surveys using the V1-V3 region and produce amplicons with lower error rates (Kozich et al., 2013; Zeeuwen et al., 2017). Negative controls processed as above showed no PCR band on an agarose gel. We also used water and reagent negative controls and mock communities (i.e., reference samples with a known composition; *Cutibacterium* was not included in the mock community) to detect contaminating microbial DNA within reagents and the measure sequencing error rate. We did not find evidence of contamination and our sequencing error rate was very low (0.0038%).

Since student samples were collected and sequenced every year for a class project (Pérez-Losada et al., 2020), sequences for the current study were generated in four consecutive runs. To account for possible batch effects, samples were processed using the same molecular protocols and handled by the same technician. Moreover, ten controls from previous years were always re-sequenced in subsequent years to validate microbial composition and structure using indices and statistical tests described below. Sequence files and associated metadata and BioSample attributes for all samples used in this study have been deposited in the NCBI (PRJNA988281). Metadata and ASV abundances with corresponding taxonomic classifications are presented in Supplementary Tables S1, S2, respectively.

### 2.4. Microbiome analyses

16S rRNA–V4 amplicon sequence variants (ASV) in each sample were inferred using the DADA2 version 1.18 (Callahan et al., 2016). Reads were filtered using standard parameters recommended by the authors (no uncalled bases, maximum of 2 expected errors and truncating reads at a quality score of 2). Forward and reverse reads were trimmed after 240 and 150 bases, respectively, merged and chimeras identified. Taxonomic assignment was performed against the SILVA v138.1 database using the RDP naive Bayesian classifier (Wang et al., 2007; Quast et al., 2013). ASV sequences (~250 bp) were aligned in MAFFT (Katoh and Standley, 2013) and FastTree, which implements an approximate maximum likelihood search strategy, was used to estimate phylogenetic relationships among ASVs (Price et al., 2010). The resulting ASV tables and phylogenetic tree were imported into phyloseq (McMurdie and Holmes, 2013) for further analysis. We normalized our samples using the negative binomial distribution as recommended by McMurdie and Holmes (2014) and implemented in the Bioconductor package DESeq2 (Love et al., 2014). This approach simultaneously accounts for library size differences and biological variability and has increased sensitivity if groups include less than 20 samples (Weiss et al., 2017). Alpha-diversity was estimated using Chao1 richness and Shannon, ACE, and phylogenetic diversity (PD) indices. Beta-diversity was estimated using phylogenetic Unifrac (unweighted and weighted), Jensen-Shannon divergence and Chao distances, and dissimilarity between samples was explored using principal coordinates analysis (PCoA).

Differences in taxonomic composition (phyla and genera) and alpha-diversity indices between skin regions (predictors) were assessed using linear mixed-effects (LME) models analysis, as implemented in the lmer4 R package (Bates et al., 2015), to account for non-independence of subjects (random effect). When assessing differences between sampling years for each skin region (i.e., independent subjects), we used linear models. We also included age, ethnicity, and gender as covariables in all our analyses as well as sampling year in the LME models. Beta-diversity indices were compared using permutational multivariate analysis of variance (adonis), as implemented in the Vegan R package (Dixon, 2003), while also accounting for covariables as indicated above.

Bacterial functional profiles were assessed using metabolic pathways predicted in PICRUSt2 (Douglas et al., 2020). We followed the standard pipeline recommended by the authors. Predicted sample gene family profiles were collapsed using the Kyoto Encyclopedia of Genes and Genomes (KEGG) Pathway metadata (Kanehisa et al., 2019). Pathway abundances were normalized using the negative binomial distribution (McMurdie and Holmes, 2014). As above, LME analysis was then used to identify differentially abundant metabolic pathways across body regions while accounting for random effects (subjects) and covariables (subjects age, ethnicity, and gender).

We applied the Benjamini-Hochberg method at alpha = 0.05 to correct for multiple hypotheses testing (Cook, 1977; Benjamini and Hochberg, 1995). All the analyses were performed in R (Team RDC, 2008) and RStudio Team (RStudio, 2015). All data files used in this study can be found in GitHub: https://github.com/mlosada323/skin-microbiome.

## 3. Results

### 3.1. Taxonomic diversity

We sampled skin swabs from a cohort of 129 adult participants (students – both undergraduate and graduate) from George Washington University, Washington, DC (Supplementary Table S1). They belonged to five main ethnicities, White (50.4%), Asian (30.2%), Black (11.6%,), Hispanic (5.4%) and Eurasian (2.4%); their mean age was 24.4 ± 7.6 years and 69.8% were female. We sequenced the V4 region of the 16S rRNA gene (~250 bp) to characterize the microbiome of five skin regions in each participant (645 skin samples). ASV singletons and samples with <1,045 reads were eliminated from further analysis, rendering a final dataset of 579 individual samples distributed across the five targeted skin regions: BB (belly button) (121 samples), BE (behind both ears) (121 samples), BT (between toes of both feet) (127 samples), CA (both calves) (104 samples) and FA (both forearms) (106 samples). Within each skin region, samples were also analyzed by year: 2019 (12–13 samples), 2020 (25–32 samples), 2022 (35–43 samples) and 2023 (30–39 samples). Samples were run for each year with controls in each run and no significant differences indicative of batch effect were observed for any of the indices tested between control samples across sequencing runs (see also Supplementary Figure S1).

The 579 samples analyzed after quality control included only 1,395 Archaea reads corresponding to 43 ASVs; this is not surprising given its low representation in the human skin (Probst et al., 2013; Oh et al., 2014). The skin bacteriome, however, accounted for 16,930,379 reads, ranging from 1,045 to 138,993 sequences per sample (mean = 29,240.7) and was comprised of 8,628 ASVs (Supplementary Table S2).

**Figure 1:**
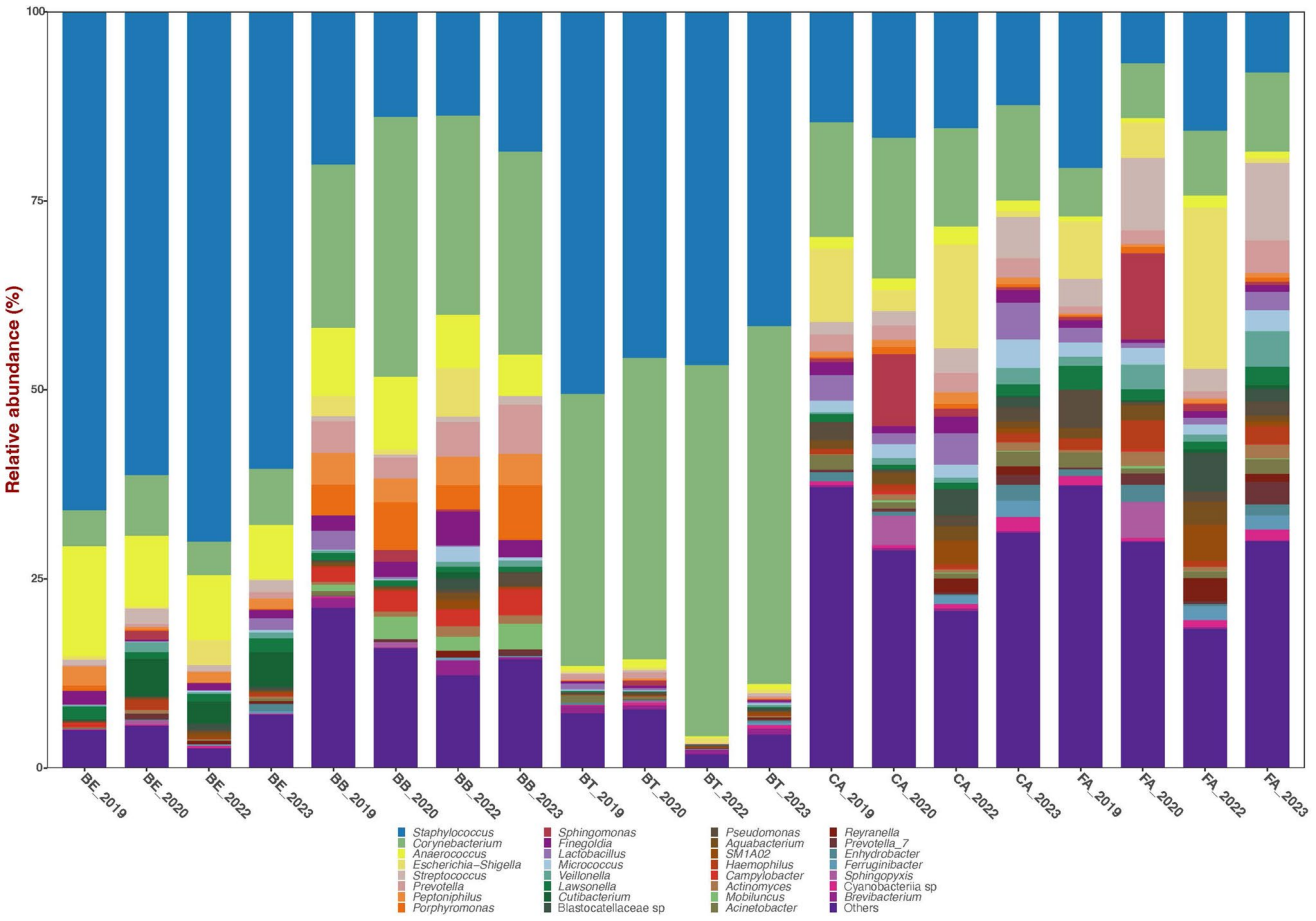
Bar plots of mean relative proportions of the top bacterial genera in the skin bacteriomes of 129 adults grouped by skin region and year. BB, belly button; BE, behind both ears; BT, between toes of both feet; CA, both calves; and FA, both forearms. Microenvironments: oily (BE), moist (BB + BT) and dry (CA + FA).

The bacterial sequences across all 579 filtered samples were classified into four dominant (>1% abundance) Phyla: Actinobacteriota (29.1%), Bacteroidota (5.2%), Firmicutes (51.1%), and Proteobacteria (10.6%). The nine most dominant genera detected across all samples were: *Staphylococcus* (35.7%), *Corynebacterium* (22.6%), *Anaerococcus* (4.9%), *Escherichia-Shigella* (3.5%), *Prevotella* (2.0%), *Streptococcus* (1.9%), *Peptoniphilus* (1.6%), *Porphyromonas* (1.6%), and *Finegoldia* (1.4%) (Figure 1).

Bacterial phyla relative abundance varied across skin regions (Table 1) and microenvironments (oily, moist and dry). Belly button (BB) and between toes (BT) (moist microenvironment) were mainly comprised of Actinobacteriota and Firmicutes; behind ears (BE) (oily microenvironment) was dominated by Firmicutes; and calves (CA) and forearms (FA) (dry microenvironment) included a combination of those three phyla. Bacterial genera relative abundance also varied across skin regions and microenvironments (Figure 1; Table 1); BB and BT (moist microenvironment) were dominated by *Staphylococcus* and *Corynebacterium*, BE (oily microenvironment) by *Staphylococcus* and *Anaerococcus*, while CA and FA (dry microenvironment) showed a high proportion of *Staphylococcus* and *Corynebacterium*, but also of *Streptococcus* and *Escherichia-Shigella. Anaerococcus* was also highly abundant in BB.

**Table 1:** Mean relative abundances (%) of dominant (>1% abundance in at least one skin region) bacterial phyla and genera in the skin bacteriomes of 129 adults grouped by skin region and microenvironment.

	BE	BB	BT	CA	FA	Oily	Moist	Dry
Phylum								
Firmicutes	85.5	43.3	51.4	35.6	35.4	82.7	44.8	34.0
Actinobacteriota	9.5	44.7	46.8	29.1	22.5	9.7	41.8	23.4
Proteobacteria	3.6	4.5	1.2	23.3	28.2	5.3	3.9	28.1
Bacteroidota	0.8	5.0	0.3	6.2	7.5	1.2	6.3	6.9
Genus								
*Staphylococcus*	70.9	25.9	50.0	17.3	14.9	67.4	31.1	15.1
*Corynebacterium*	5.8	39.5	44.4	16.3	10.7	5.7	36.4	12.3
*Anaerococcus*	10.8	8.2	0.3	2.1	1.6	9.8	4.1	1.8
*Escherichia-Shigella*	0.6	1.2	0.2	5.2	7.1	1.4	1.4	9.6
*Streptococcus*	0.8	0.6	0.1	3.4	8.3	1.1	0.3	5.5
*Prevotella*	0.2	1.9	0.1	2.5	2.3	0.4	2.7	2.3
*Sphingomonas*	0.2	0.2	0.1	2.3	2.0	0.2	2.9	0.5
*Lactobacillus*	0.2	0.3	0.1	4.3	1.4	0.3	0.2	2.9
*Micrococcus*	0.1	1.4	0.1	2.5	2.1	0.2	0.5	1.9
*Finegoldia*	0.9	2.6	0.1	1.5	0.8	1.1	1.7	1.3
*Peptoniphilus*	1.3	1.7	0.0	1.1	0.5	1.6	1.9	0.9
*Porphyromonas*	0.3	2.5	0.0	0.6	0.4	0.2	2.9	0.5

BB, belly button (121 samples); BE, behind both ears (121 samples); BT, between toes of both feet (127 samples); CA, both calves (104 samples); and FA, both forearms (106 samples).

All the four and 12 most abundant phyla and genera, respectively, showed significant differences (*p* < 0.0001; LME test) in their mean relative proportions across all skin regions and microenvironments, except for *Finegoldia* (Supplementary Table S3). Skin region pairwise comparisons of the most abundant phyla and genera showed the highest numbers of significant differences (LME test) between CA or FA and other skin regions, while CA – FA showed the lowest number of significantly different taxa (Supplementary Table S3). Accordingly, microenvironment pairwise comparisons of the most abundant phyla and genera showed the highest numbers of significant differences (LME test) between dry and other microenvironment, while moist-oily showed the lowest number of significantly different taxa (Supplementary Table S3).

BB, BE, BT, FA, and CA samples had 586, 613, 777, 1,703, and 2,366 unique ASVs, respectively (Supplementary Figure S2). The five groups shared 455 unique ASVs, while paired groups shared a variable number, ranging from 437 (CA and FA) to 21 ASVs (BB and BE). Only two ASVs (ASV1 and ASV2) of the genera *Staphylococcus* and *Corynebacterium* comprised the skin core bacteriome of all samples (90% prevalence) and accounted for 32 and 12.8% of the total reads, respectively. These same genera have also been assigned to the skin core in other studies of different cohorts (Costello et al., 2009; Wang et al., 2021) and may represent the more stable and consistent bacterial members of the skin bacteriota (Backhed et al., 2012; Shade and Handelsman, 2012).

**Figure 2:**
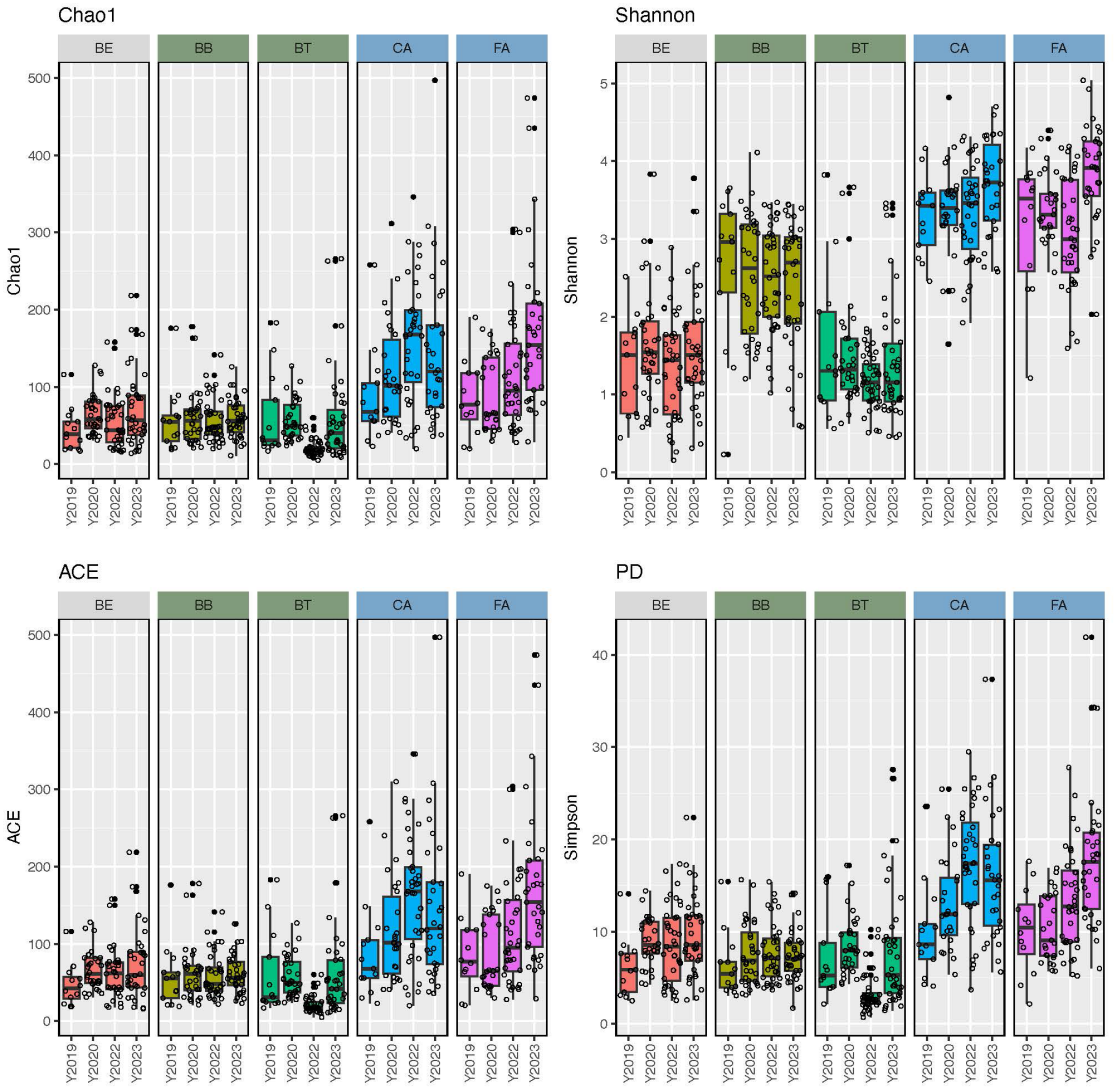
Alpha-diversity estimates (Chao1, Shannon, ACE, and phylogenetic diversity) of the skin bacteriomes of 129 adults grouped by skin region and year. BB, belly button; BE, behind both ears; BT, between toes of both feet; CA, both calves; and FA, both forearms. Oily, moist and dry regions are colored in light gray, green and blue, respectively.

Alpha-diversity indices (Chao1, Shannon, ACE, and PD) of microbial community richness and evenness varied significantly (*p* < 0.0001) among skin regions and microenvironments (Figure 2; Supplementary Tables S4, S5). CA and FA showed the highest diversity for all indices, while BT showed the lowest and BB and BE showed intermediate values. Accordingly, the dry microenvironment showed the highest diversity for all indices, the moist microenvironment displayed the lowest for all indices but Shannon, and the oily microenvironment showed intermediate values for all alpha-diversity indices but Shannon, where it showed the lowest (Supplementary Table S4). All skin region pairwise comparisons of alpha-diversity indices involving FA or CA and any other region were significantly different (*p* < 0.0001); while most of the other skin region pairwise comparisons were not significantly different (Supplementary Table S5). Similarly, all of the microenvironment pairwise comparisons of alpha-diversity indices involving dry and any other microenvironment resulted significant differences (*p* < 0.0001); while moist-oily comparisons were significant for Shannon and PD (*p* ≤ 0.0013), but were not for Chao1 and ACE (Supplementary Table S5).

**Figure 3:**
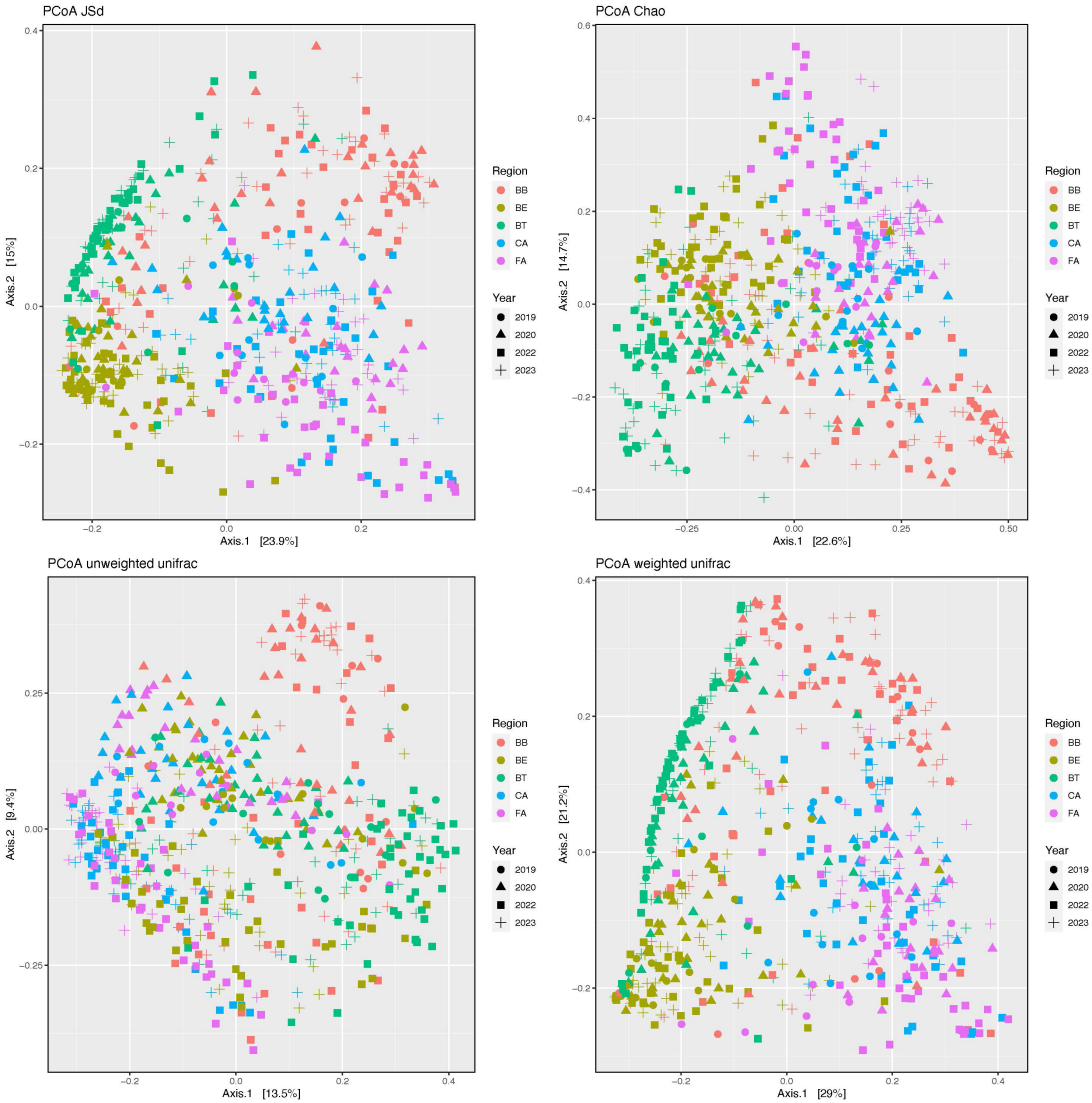
Principal coordinates analysis (PCoA) plots of beta-diversity distances (JSd, Chao and Unifrac) of the skin bacteriomes of 129 adults grouped by skin region and year. BB, belly button; BE, behind both ears; BT, between toes of both feet; CA, both calves; and FA, both forearms. Microenvironments: oily (BE), moist (BB + BT) and dry (CA + FA).

Our PCoAs (Figure 3) of beta-diversity estimates showed segregation of the skin bacteriomes across regions and microenvironments for all distances (JSd, Chao, and Unifrac). The adonis analyses detected significant differences (*p* < 0.0001) in community structure (beta-diversity) across all regions and microenvironments and for all pairwise comparisons of both factors (Supplementary Table S5).

We also compared alpha-diversity indices across years (2019, 2020, 2022, and 2023) within skin regions (Supplementary Table S6). BB showed no significant differences for any of the four indices compared (lm tests), while BE only showed significant differences in PD for 2019–2023; the other three skin regions (BT, CA, and FA) showed significant differences in alpha-diversity for different year combinations except 2019–2020. Similarly, beta-diversity analyses across years within skin regions detected significant differences in microbial structure for all regions and indices compared, except for the Unifrac weighted in BB and BT (Supplementary Table S6). Since we carried out a cross-sectional study (i.e., participants were only sampled at a single timepoint), our statistical analyses cannot separate interpersonal from time-related variation, so we can only conclude that skin bacterial stability may be region and subject dependent, as previously suggested (Costello et al., 2009; Grice et al., 2009; Oh et al., 2016).

Finally, we assessed variation in bacterial composition (phyla and genera) and microbial community richness and evenness in relation to age, ethnicity, and gender using LME models, but no significant differences were observed, suggesting a greater impact of regionalization of the skin microbiome.

### 3.2. Functional diversity

Our functional analysis in PICRUSt2 showed 36 to 397 metabolic pathways significantly (*p* < 0.05) differentially expressed after FDR correction between pairs of skin regions (Supplementary Figure S3; Supplementary Table S7). As described above for taxonomic profiles, the highest number of significantly different pathways (LME test) was observed between CA or FA and other skin regions (354 to 397), BE – BB – BT showed 253 to 301 significant pathways, while CA – FA showed only 36 significantly pathways. Accordingly, the highest number of significantly different metabolic pathways (LME test) were also observed between the dry microenvironment and the moist and oily microenvironments (372 and 400 metabolic pathways, respectively), while moist-oily showed 261 significant pathways. When pathways were grouped in five basic KEGG categories (Genetic Information Processing, Environmental Information Processing, Cellular Processes, Metabolism and Organismal Systems) all of them resulted in significant differences for both biogeographic factors (*p* < 6.1E-12).

## 4. Discussion

We characterized the taxonomic and functional bacterial diversity of 579 skin samples (129 healthy adults) representing five skin regions (belly button, behind ears, between toes, calves and forearms) and three different skin habitats or microenvironments (sebaceous, dry and moist) collected once in 2019, 2020, 2022, and 2023.

### 4.1. Taxonomic diversity

The skin bacteriome was dominated by the phylum Firmicutes (51.1%), followed by Actinobacteriota (29.1%), Proteobacteria (10.6%), and Bacteroidota (5.2%). These same four phyla have also been found to be predominant in other studies of the skin microbiome, although with different abundances (Grice et al., 2009; Oh et al., 2014). *Staphylococcus* (35.7%) and *Corynebacterium* (22.6%) were the most abundant genera across all samples, while none of the other predominant genera had a mean relative abundance >5%. These two genera are common members of the human epidermis (Grice et al., 2009; Human Microbiome Project, 2012; Oh et al., 2014; Byrd et al., 2018; Cundell, 2018; Dimitriu et al., 2019). *Cutibacterium* only accounted for 0.55% of all the reads. This could result from primer bias in our 16S sequencing protocol (Kong, 2016) or unique features of the studied cohort.

All four predominant bacterial phyla and at least eleven of the twelve predominant genera varied significantly in their mean relative abundances across skin regions and microenvironments (Figure 1; Table 1; Supplementary Table S3). CA or FA (dry microenvironment) versus other skin regions or microenvironments showed the highest number of significant taxon differences (LME test), while CA – FA and moist-oily showed the lowest (Supplementary Table S3). The human epidermis comprises diverse microenvironments that vary in ultraviolet light exposure, pH, temperature, moisture, sebum content, and topography (Grice et al., 2009; Grice and Segre, 2011; Byrd et al., 2018). Based on these characteristics, the five skin regions sampled here can be grouped into three broad categories: sebaceous or oily (BE); moist (BB and BT) and dry (CA and FA). These habitats or microenvironments vary in abundance of sweat and sebaceous glands and hair follicles. Sweat glands are more abundant in moist regions, so they evaporate more water, which in turn also acidifies the skin, making conditions unfavorable for the growth and colonization of certain microorganisms (Grice and Segre, 2011). Additionally, sweat contains antimicrobial molecules that inhibit microbial colonization (Gallo and Hooper, 2012). Connected to the hair follicle, sebaceous glands are denser in oily regions; they secrete lipid-rich sebum, a hydrophobic coating that lubricates and provides an antibacterial shield to hair and skin (Byrd et al., 2018). Therefore, skin microhabitat variation across regions and more broadly across microenvironments is likely responsible for the bacterial community diversity observed here; skin areas with physiologically comparable sites bear more similar bacterial communities, while those physiological distinct harbor more different bacteriomes. Other studies have also shown significant differences across these same or comparable skin regions and microenvironments, although the mean relative abundances of the bacterial taxa involved varied across studies (Costello et al., 2009; Grice et al., 2009; Oh et al., 2014). This is not surprising given the existing methodological differences across studies (Kong, 2016; Meisel et al., 2016) and the variation in the demographics (Dimitriu et al., 2019; Wang et al., 2021) and personal habits (Grice et al., 2009; Byrd et al., 2018; Cundell, 2018; Pérez-Losada et al., 2020; Skowron et al., 2021) of the cohorts studied.

Bacterial community richness and evenness (Figure 2; Supplementary Tables S4, S5) and structure (Figure 3; Supplementary Table S5) also varied significantly (*p* < 0.0001) among skin regions and microenvironments (*p* ≤ 0.0013). Dry regions (CA and FA) showed the highest within-sample diversity for all indices (twice as high in some cases), while BT showed the lowest and BB and BE showed intermediate values. The moist microenvironment (BB and BT) also showed the lowest alpha-diversity for most indices. As for beta-diversity, BB showed the least similarity among samples, followed by CA and FA (dry microenvironment), while the most similar regions were BE and BT. Previous studies (Costello et al., 2009; Grice et al., 2009; Oh et al., 2014) have also shown that bacteriomes from dry skin sites display the highest richness and evenness; they also showed the lowest similarity (beta-diversity) for interdigital web spaces and navel (Grice et al., 2009), forearms (Costello et al., 2009) or sebaceous sites in general (Oh et al., 2014). Several intrinsic and extrinsic factors alone or combined may explain these differences in microbial diversity; as indicated above, dry skin regions present more favorable conditions for the growth and survival of bacteria (Grice and Segre, 2011; Gallo and Hooper, 2012). Additionally, external environmental conditions (temperature, humidity, and sunlight – UV radiation) can also alter the bacteriomes of exposed skin regions like forearms and calves differently than those of covered regions (Boxberger et al., 2021; Skowron et al., 2021). Moreover, washing habits or skin care products may also disrupt bacterial communities differently and play a role here, since one could expect that some areas like calves and forearms are washed and lubricated more often than others (between toes or navels) (Fierer et al., 2008; Grice and Segre, 2011; Two et al., 2016; Cundell, 2018; Pérez-Losada et al., 2020; Boxberger et al., 2021; Skowron et al., 2021). Finally, gender, age, and ethnicity have also been suggested as primary or secondary contributing factors to skin diversity in other studies (Ying et al., 2015; Li et al., 2019; Skowron et al., 2021); however, we have not detected significant differences associated to these three factors in our cohort.

### 4.2. Functional diversity

Contrary to previous studies (Human Microbiome Project, 2012) which reported that most metabolic pathways are evenly distributed across body sites, we detected significant variation in functional diversity across skin regions and microenvironments (Supplementary Figure S3; Supplementary Table S7). As described above for taxonomic composition and diversity, pairwise comparisons involving dry regions (CA and FA) and microenvironments showed the highest differences in metabolism (83–94% pathways) when compared to moist and sebaceous skin regions (59–71%). A metagenomic analyses of the skin microbiome (Oh et al., 2014) showed that 88% of the metabolic modules were also differentially abundant in at least one skin microenvironment, hence suggesting that functional capacity is driven primarily by biogeography, as reported here.

### 4.3. Limitations

Metataxonomic studies like this suffer from the inherent limitations (e.g., marker validation, technical biases and limited taxonomic resolution) (Odom et al., 2023) of collecting sequence data from a single partial gene target (16S rRNA). Nevertheless, the inferred composition of the skin bacteriomes in this study is similar to those described in previous metataxonomic and metagenomic studies of the skin. Additionally, our functional analysis (PICRUSt2) of 16S data can only predict metabolic pathways that need to be confirmed using genomic data. Although we collected a large number of samples (579 after data cleaning), the number of regions surveyed is small and their distribution across regions and years is uneven, which may also impact our statistical results despite their high significance. Finally, our sampling is not longitudinal (i.e., we sampled different participants every year rather than the same individuals at multiple timepoints), hence we cannot study skin bacterial temporal diversity and dynamics or separate interpersonal from time-related variation. We hope we can address and correct some of these issues in future studies.

## 5. Conclusion

The taxonomic and functional diversity of the skin bacteriome in healthy subjects is poorly understood. We analyzed an adult cohort of 129 individuals (579 samples) sampled once at four different years to generate insights into these issues. We showed that bacterial diversity varies spatially across skin regions (belly button, behind ears, between toes, calves, and forearms) and microenvironments (dry, moist, and sebaceous). This study provides a reference (i.e., normal or healthy skin bacteriome) for other studies that examine the role of bacterial communities in skin diseases (i.e., dysbiosis) and the impact of internal and external factors on the skin bacteriome.

## Data availability statement

The data presented in this study are deposited in the NCBI repository under accession number PRJNA988281.

## Ethics statement

The studies involving humans were approved by The George Washington University Committee on Human Research, Institutional Review Board, IRB# 180703. The studies were conducted in accordance with the local legislation and institutional requirements. The participants provided their written informed consent to participate in this study.

## Author contributions

MP-L: Conceptualization, Data curation, Formal analysis, Funding acquisition, Investigation, Methodology, Project administration, Resources, Software, Supervision, Validation, Visualization, Writing – original draft, Writing – review & editing. KC: Conceptualization, Funding acquisition, Project administration, Writing – review & editing.

## Funding

The author(s) declare financial support was received for the research, authorship, and/or publication of this article. This project was partially supported by a Fellowship Award from the GWU Milken Institute School of Public Health’s Academy of Master Teachers and grants from the Milken Institute School of Public Health Pilot Fund Program.

## Conflict of interest

The authors declare that the research was conducted in the absence of any commercial or financial relationships that could be construed as a potential conflict of interest.

The author(s) declared that they were an editorial board member of Frontiers, at the time of submission. This had no impact on the peer review process and the final decision.

## Publisher’s note

All claims expressed in this article are solely those of the authors and do not necessarily represent those of their affiliated organizations, or those of the publisher, the editors and the reviewers. Any product that may be evaluated in this article, or claim that may be made by its manufacturer, is not guaranteed or endorsed by the publisher.
