# Supplementary figures and images for: Spatial diversity of the skin bacteriome
Source: Front Microbiol. 2023 Sep 19;14:1257276. doi: 10.3389/fmicb.2023.1257276 (PMC10546022; doi:10.3389/fmicb.2023.1257276)

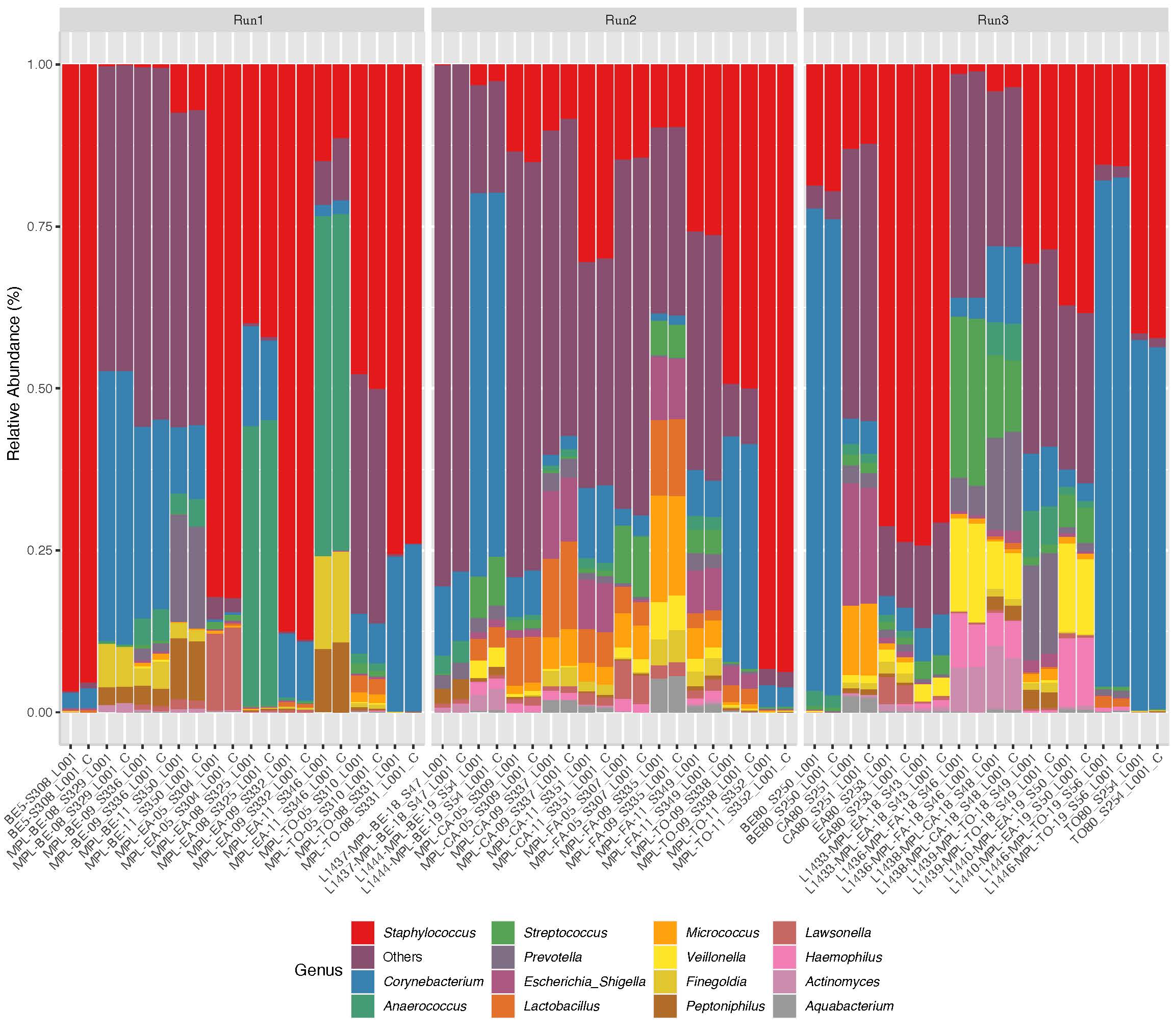

Supplement: Supplementary file 1 [file Image_1.JPEG]

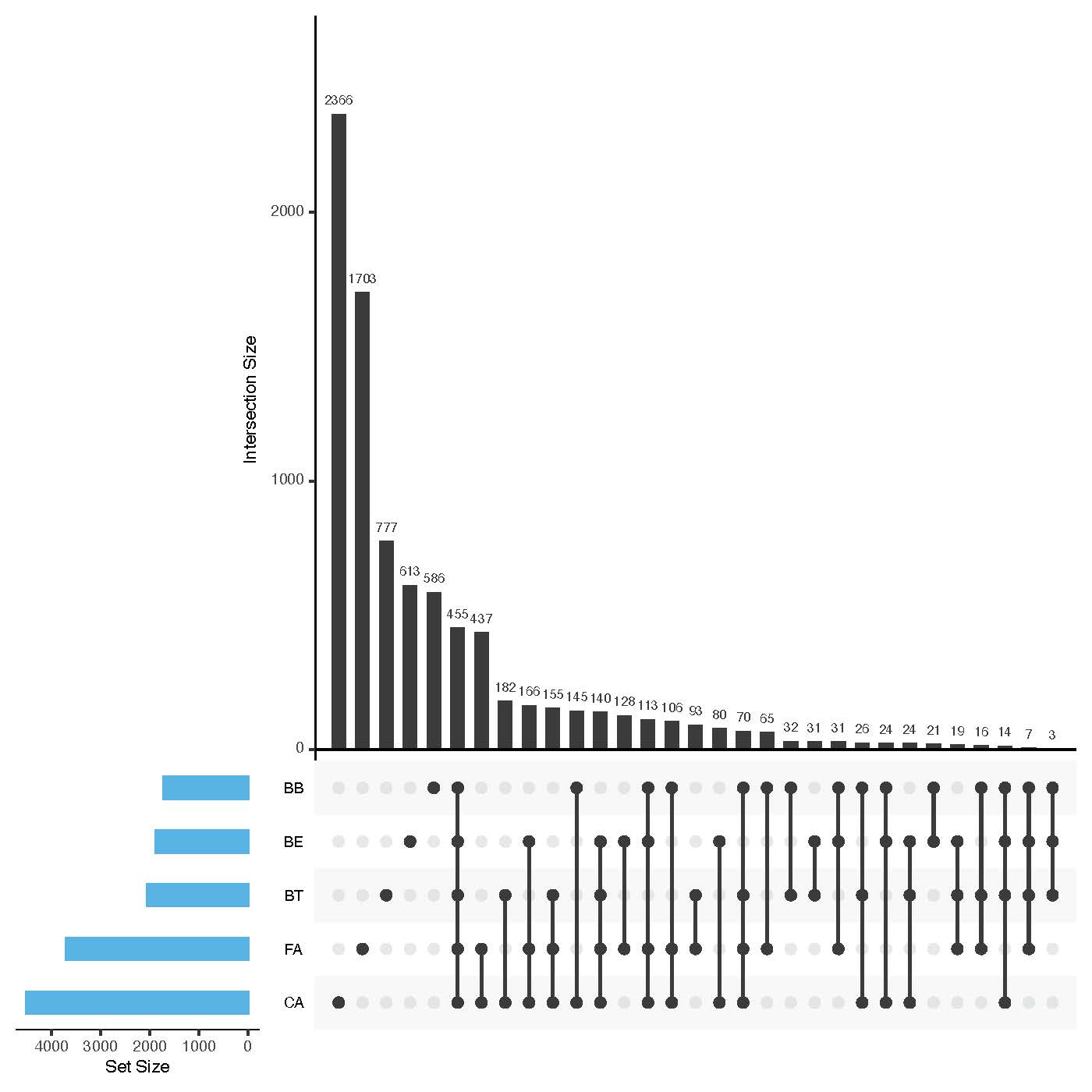

Supplement: Supplementary file 2 [file Image_2.JPEG]

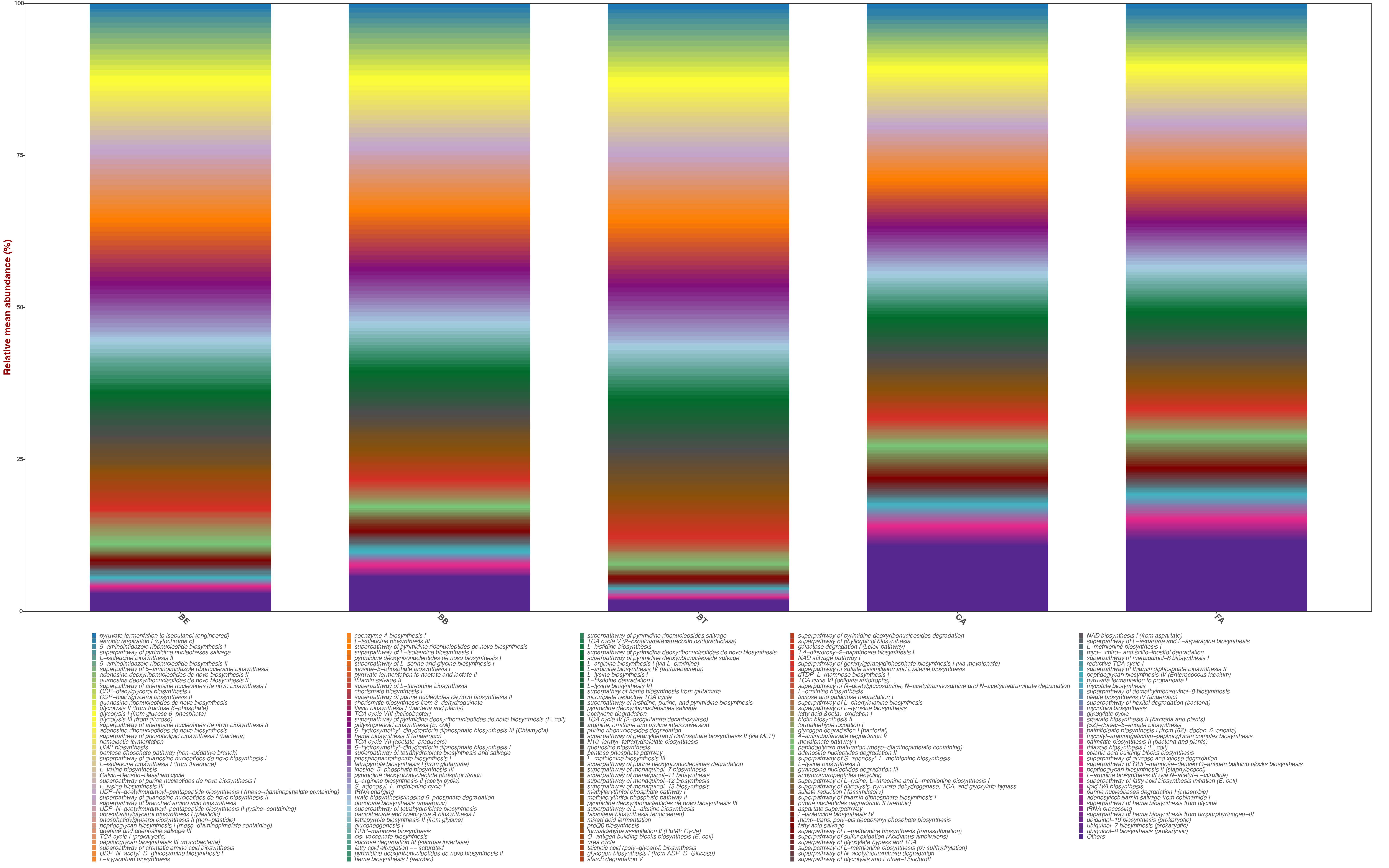

Supplement: Supplementary file 3 [file Image_3.JPEG]
